# Novel multiplex PCR-SSP method for centromeric *KIR* allele discrimination

**DOI:** 10.1038/s41598-018-33135-1

**Published:** 2018-10-05

**Authors:** Jean-Benoît Le Luduec, Anupa Kudva, Jeanette E. Boudreau, Katharine C. Hsu

**Affiliations:** 1 Immunology Program, Sloan-Kettering Institute for Cancer Research, New York, NY USA; 2 Department of Pediatrics, Memorial Sloan Kettering Cancer Center, New York, NY USA; 3 Department of Medicine, Weill Cornell Medical College, New York, NY USA; 4 Department of Medicine, Memorial Sloan Kettering Cancer Center, New York, NY USA; 5 Present Address: Departments of Pathology and Microbiology & Immunology, Dalhousie University, Halifax, Canada

**Keywords:** Immunogenetics, Innate lymphoid cells

## Abstract

Allelic diversity of the KIR2DL receptors drive differential expression and ligand-binding affinities that impact natural killer cell function and patient outcomes for diverse cancers. We have developed a global intermediate resolution amplification-refractory mutation system (ARMS) PCR-SSP method for distinguishing functionally relevant subgroups of the *KIR2DL* receptors, as defined by phylogenetic study of the protein sequences. Use of the ARMS design makes the method reliable and usable as a kit, with all reactions utilizing the same conditions. Six reactions define six subgroups of *KIR2DL1*; four reactions define three subgroups of *KIR2DL2*; and five reactions define four subgroups of *KIR2DL3*. Using *KIR* allele data from a cohort of 426 European-Americans, we identified the most common *KIR2DL* subtypes and developed the high-throughput PCR-based methodology, which was validated on a separate cohort of 260 healthy donors. Linkage disequilibrium analysis between the different *KIR2DL* alleles revealed that seven allelic combinations represent more than 95% of the observed population genotypes for *KIR2DL1/L2/L3*. In summary, our findings enable rapid typing of the most common *KIR2DL* receptor subtypes, allowing more accurate prediction of co-inheritance and providing a useful tool for the discrimination of observed differences in surface expression and effector function among NK cells exhibiting disparate *KIR2DL* allotypes.

## Introduction

As key members of the innate immune response, natural killer (NK) cells survey surrounding cells, discriminating damaged or infected cells from healthy cells, in part via receptor recognition of altered self-MHC on damaged cells^1^. This process, termed “education” or “licensing” is enabled through interactions between inhibitory receptors on NK cells with “self” MHC, that permit cytotoxic granule release for target cell killing, but also inhibition of the NK cell upon binding to cognate MHC. In humans, the principal receptors mediating education are the polygenic, polymorphic inhibitory killer cell immunoglobulin-like receptors (KIR), which recognize antigens presented by HLA-A, -B, and –C molecules^2^.

The *KIR2DL* receptors exclusively recognize HLA-C molecules: *KIR2DL1* recognizes HLA-C allotypes characterized by Lys80 (collectively referred to as HLA-C group 2); *KIR2DL3* recognizes predominantly HLA-C allotypes characterized by Asn80 (HLA-C group 1); while *KIR2DL2* recognizes members of both HLA-C groups 1 and 2^2^. Between the *KIR2DL* receptors and their specificities, nearly all HLA-C allotypes have a cognate inhibitory KIR. Additional inhibitory KIR molecules include KIR3DL1, which recognizes the Bw4 epitope exhibited by some HLA-A and HLA-B allotypes, and KIR3DL2, which recognizes the HLA-A3, HLA-A11, and HLA-B27 proteins^2,3^.

Significant diversity exists from individual to individual both at the *KIR* gene content and allele level. Some patterns of genetic combination are well-recognized and have led to the designation of the canonical *KIR* haplotype-A, characterized by gene content as presence of the centromeric *KIR2DL3* and *KIR2DL1* and the telomeric *KIR3DL1*, in the absence of all activating *KIR*, with the exception of the telomeric *KIR2DS4*. The remaining haplotypes collectively comprise the *KIR* B-haplotypes, exhibiting differing numbers and types of activating *KIR* in the centromeric or telomeric portions^4–6^. Clinical consequences of *KIR* diversity, even at the level of gene content, have provided some clues to the importance of differentially educated NK cells in control of viral infection, such as hepatitis C^7^, HIV^8^ and hematologic malignancy^9,10^.

Allelic polymorphism further diversifies the educational breadth of the NK repertoire. It is increasingly clear that different alleles of the same receptor, *KIR3DL1*, exhibit different surface expression properties and affinities for the same HLA ligand^11–13^, leading to substantial variations in NK education and sensitivity to inhibition^11,12^. These findings have enabled a more intricate understanding of NK education, again with important implications in viral control^11,14^ and malignancy^15^. Whether allele subtype variation for KIR2DL similarly impacts NK cell function and disease outcomes has not been extensively studied. However, it is known that the KIR2DL receptors are highly polymorphic, and that allelic variation may influence cell surface expression^16,17^, as well as avidity and specificity for HLA-C ligands^18,19^, potentially leading to benefits in infectious disease^7,20^.

While the clinical ramifications of allele-driven *KIR* diversity continue to emerge, a lack of straightforward technology to discriminate *KIR* content at the allele level has hampered large-scale clinical studies. Next-generation sequencing technology for *KIR* allele typing remains investigational^21^ or out of practical reach for research laboratories. We previously reported an accessible multiplex PCR assay for the cost-effective discrimination of *KIR3DL1* alleles, and we have employed this assay in a large retrospective analysis of hematopoietic cell transplantation patients to demonstrate the clinical impact of functional *KIR* subtyping^15,22^. We now present a similar approach for the centromeric inhibitory KIR genes *KIR2DL1*, *KIR2DL2*, and *KIR2DL3*, identifying the nucleotide sites potentially important for functional discrimination among receptor alleles and devising an amplification-refractory mutation system (ARMS) PCR-SSP typing methodology. We anticipate that functional classification of the centromeric inhibitory *KIR*, as has been done from the telomeric *KIR3DL1/S1*, will broaden our understanding of how these alleles influence human health and disease.

## Results

### *KIR2DL1* allele typing

**Figure 1:**
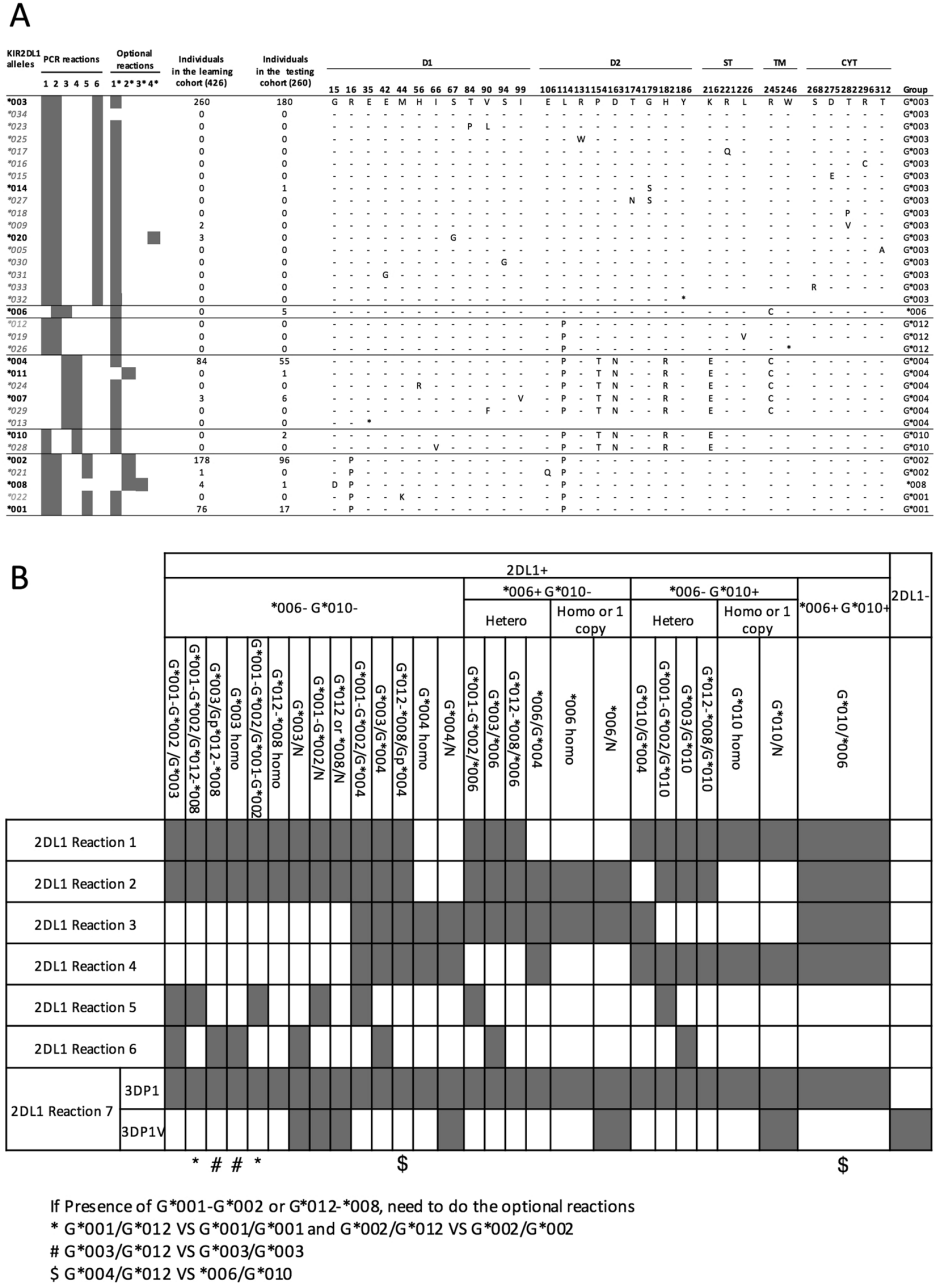
*KIR2DL1* allele typing method. (**A**) Alignment of the amino acid sequences of the 34 known *KIR2DL1* allelic variants. A dash indicates identity with the consensus KIR2DL1*003, and an (*) indicates a stop codon. Structural domains are indicated: Ig-like domains (D1 and D2), stem domain (ST), transmembrane domain (TM), and cytoplasmic domain (CYT). Six PCR reactions separate the six subgroups identified by phylogenetic analysis. Four additional PCR reactions separate alleles within subgroups. Frequencies of the alleles present in the learning cohort of 426 individuals and in the testing cohort of 260 individuals are indicated. The group identification number for each allele is indicated. Alleles identified by PCR are in bold black font, and alleles that were not tested are in gray italics. (**B**) *KIR2DL1* PCR interpretation guide. PCR profiles marked by *, #, or $ are similar and require a higher resolution of genotyping using supplemental reactions.

We examined the *KIR* alleles previously identified by sequence-based typing in a cohort of 426 healthy individual donors^23^. Of the 34 known *KIR2DL1 alleles* (EMBL-EBI IPD KIR), four (*001, *002, *003, *004) occurred frequently, with a presence for each allele in 18% or more of the individuals in the cohort (Fig. 1A). Five other alleles (*007, *008, *009, *020, *021) were found in fewer than 1% of all individuals. The remaining alleles were not identified in the cohort. Allelic distribution and phylogenetic analysis (Supplemental Fig. 1A) identified six non-overlapping groups. Six distinct ARMS PCR reactions specific for these six groups and four supplemental reactions to further discriminate alleles present within the group (Table 1) were optimized using DNA from 178 of the original 426 donors. We designed an additional reaction to identify the pseudogene *KIR3DP1* and its variants (*KIR3DP1V*), the latter characterized by the presence of an exon 2. The presence of *KIR3DP1V* on the chromosome 19 is associated with the absence of *KIR2DL1* on the same haplotype^5,24,25^. Detection of *KIR3DP1* and *KIR3DP1V* can therefore be used to estimate *KIR2DL1* copy number.

**Table 1:** KIR2DL PCR primers and target site positions.

Reaction	Primers name	Nucleotide targeted	Sequence Primers	Size amplicon (bp)
Control	ControlF	NA	CCAAGCCCAACCTTAAGAAGAAAATTGGAG	813
	ControlR	NA	CCAAACCCACGGTACGCATGGGAACACTGC	
2DL1 Reaction 1	2DL1R1F	3680	AGAGATAAGACACCAGGAAGGGGAAGCCCG	388
	2DL1R1R	4011	TGTCCAGAGGGTCACTGGGAGCTGACTC	
2DL1 Reaction 2	2DL1R2F	5499	GAGAGAGAGAGAGAGAGAGCATTAGGTCATAGTA	383
	2DL1R2R	5820	TGACTTTGACCACTCGTATGGAGAGTCTT	
2DL1 Reaction 3	2DL1R3F	13420	ATCCTCTTCATCCTCCTCTTCTTTCTCCTTCACT	252
	2DL1R3R	13609	CAGTTCAGAATCAGGCAACGGTCTGTGAAT	
2DL1 Reaction 4	2DL1R4F	5499	GAGAGAGAGAGAGAGAGAGCATTAGGTCATAGGA	297
	2DL1R4R	5735	TGGCCTGGAATGTTCCGTTGACCTTGCT	
2DL1 Reaction 5	2DL1R5F	3790	AACCTTCCCTCCTGGCCCACCCAGGTAC	278
	2DL1R5R	4011	GATGTCCAGAGGGTCACTGGGAGCTGACGC	
2DL1 Reaction 6	2DL1R6F	5616	ATATGAGAAACCTTCTCTCTCAGCCCAGTT	202
	2DL1R6R	5761	GTGGGTGGCAGGGCCCAGAGGAAAGTAA	
2DL1 Reaction 7	3DP1F	NA	ACGTGTTGTGAGTTGGTCATAGTGA	649
	3DP1VF	NA	AAGTGGAAATGGGAGAATCTTCTGAC	382
	3DP1R	NA	GCCCTCTGACCTGTGACCATGATC	
2DL1 Optional 1	2DL1O1F	71	GTTGGTCATAGTGAAGGACACTAGGTGTCAAATTCTATC	274
	2DL1O1R	281	TCACCAACACACGCCATGCTGACGTC	
2DL1 Optional 2	2DL1O2F	281	CTCCGGCAGCACCATGTCGCTCTTAT	390
	2DL1O2R	620	CCGTAACTCCACCTCCAGGCCCATTA	
2DL1 Optional 3	2DL1O3F	3787	AAACCTTCCCTCCTGGCCCACCCAAA	376
	2DL1O3R	4110	CTTCCTTACAGCCACCTGGGTCTCCAGT	
2DL1 Optional 4	2DL1O4F	3942	GGGTCTCCAAGGCCAACTTCTCCATGG	222
	2DL1O4R	4110	CTTCCTTACAGCCACCTGGGTCTCCACT	
2DL2 Reaction 1	2DL2R1F	5663	TATCCAGGGAGGGGGAGGCCCATGATT	211
	2DL2R1R	5820	TGAGACAGATATGGGGTTTCCTCACCAG	
2DL2 Reaction 2	2DL2R2F	5663	TATCCAGGGAGGGGGAGGCCCATGATT	210
	2DL2R2R	5820	GAGACAGATATGGGGTTTCCTCACCCA	
2DL2 Reaction 3	2DL2R3F	13995	ACAGATGCTGCGGTAATGGACCAAGATT	309
	2DL2R3R	14249	ATCTGGACTCAGCATTTGGAAGTTCCCC	
2DL2 Reaction 4	2DL2R4F	11984	CTACTTCCAATCACCTGTGGAGATTCATG	2322
	2DL2R4R	14249	ATCTGGACTCAGCATTTGGAAGTTCCTT	
2DL2 Optional 1	2DL2O1F	3754	AACCTTCCCTCCTGGCCCACCCAGGTTC	191
	2DL2O1R	3890	CATCATGGGACCGATGGAGAAGTTGGTT	
2DL2 Optional 2	2DL2O2F	3754	AACCTTCCCTCCTGGCCCACCCAGGTAG	191
	2DL2O2R	3890	CATCATGGGACCGATGGAGAAGTTGGGT	
2DL3 Reaction 1	2DL3R1F	13892	ATGAAATGAGGGCCCAGAAGTGCCCTGT	314
	2DL3R1R	14154	GGTGTCTTGGGCCTCTGAGAAGGAC	
2DL3 Reaction 2	2DL3R2F	3825	CACAGAGAAGGGAAGTTTAAGGACACTTTGTG	399
	2DL3R2R	4168	TGTATGGCCCCTGTGTCTGTCCTTT	
2DL3 Reaction 3	2DL3R3F	9063	CTGTCTCATGTTCTAGGAAACCCTTCAAATAGTTGGGT	319
	2DL3R3R	9303	GAAGGATGTCAGATTGGCAATCATTCTTCTAGCTTGTAGGAAA	
2DL3 Reaction 4	2DL3R4F	13973	GCCTGCAGGGAACAGAACAGTGAACAAG	233
	2DL3R4R	14154	GGTGTCTTGGGCCTCTGAGAAGGCT	
2DL3 Reaction 5	2DL3R5F	3853	CCTCATTGGAGAGCACCATGATGGGGCT	430
	2DL3R5R	4222	CCTCTCTCTGGGACATGTCTGTCTGTCTGTCTGT	
2DL3 Optional 1	2DL3O1F	3708	TAGGAGTCCACAGAAAACCTTCCCTCGG	323
	2DL3O1R	3976	GAATGTCCGGACACTCTCACCTGTGACG	
2DL3 Optional 2	2DL3O2F	16795	CCCTCCATCTGGGTGCTTGTCCTAAAGGCG	213
	2DL3O2R	16949	GCGATGAAGGAGAAAGAAGAGGAGGAGGTC	
2DL3 Optional 3	2DL3O3F	17646	TGAACAAGACCCTCAGGAGGTGACATTT	169
	2DL3O3R	17761	TCATGGGCAGGAGACAACTTTGGATAT	
2DL3 Optional 4	2DL3O4F	7315	TCCTGCAATGTTGGTCAGATGTCAGGTTCG	643
	2DL3O4R	7903	AGGCCACAGGGCCCAACTCAGGTCGT	
2DL3 Optional 5	2DL3O5F	13892	ATGAAATGAGGGCCCAGAAGTGCCCTGT	278
	2DL3O5R	14111	CTCTGTGTGAAAACGCAGTGATTCAACTGTTT	
2DL3 Optional 6	2DL3O6F	13892	ATGAAATGAGGGCCCAGAAGTGCCCTGT	278
	2DL3O6R	14111	CTCTGTGTGAAAACGCAGTGATTCAACTGTTC	

Altogether, by using all eleven PCR reactions, we were able to separate ten different individual alleles or groups of alleles (*KIR2DL1*-G*001, -G*002, -G*003, -G*004, -G*012, *006, *008, *010, *011, *020) exhibited individually or in combination (Supplemental Fig. 2A,B). A few combinations of alleles, involving *KIR2DL1*-G*012 in particular, cannot be resolved with our method (Fig. 1B). It should be noted that these alleles are rare, and none of them was found in the 426 sequenced individuals.

### *KIR2DL2* allele typing

**Figure 2:**
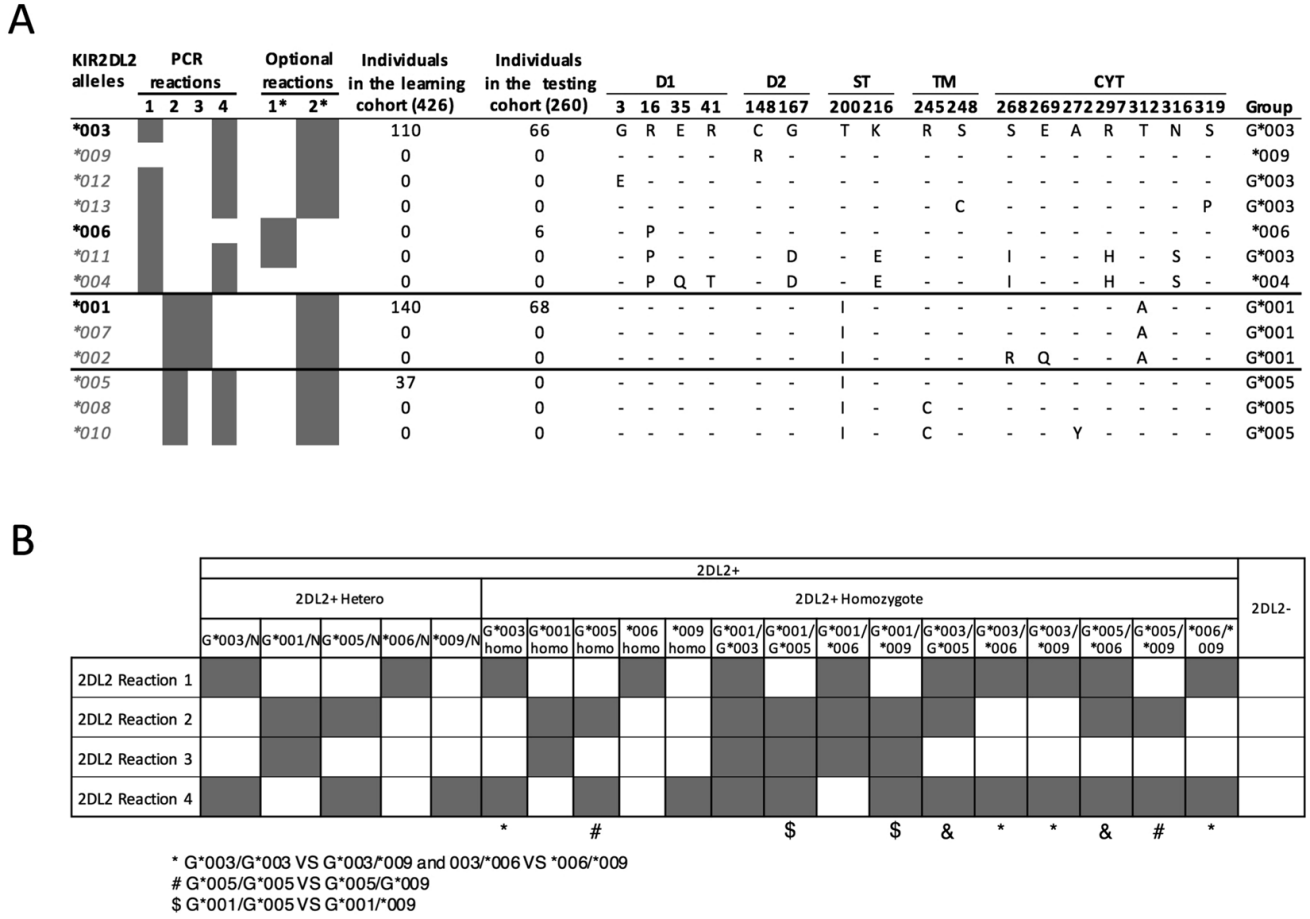
*KIR2DL2* allele typing method. (**A**) Alignment of the amino acid sequences of the 13 known *KIR2DL2* allelic variants. A dash indicates identity with the consensus KIR2DL2*003. Structural domains are indicated: Ig-like domains (D1 and D2), stem domain (ST), transmembrane domain (TM), and cytoplasmic domain (CYT). Four PCR reactions separate the three subgroups identified by phylogenetic analysis. Two additional PCR reactions separate alleles within subgroups. Frequencies of the alleles present in the learning cohort of 426 individuals and in the testing cohort of 260 individuals are indicated. The group identification of each allele is indicated. The alleles tested by PCR are in bold black font, and non-tested alleles are in gray italics. (**B**) *KIR2DL2* PCR interpretation guide. PCR profiles marked by *, #, $ or & are similar and require a higher resolution of genotyping using supplemental reactions.

Among the 13 *KIR2DL2* alleles published in EMBL-EBI IPD KIR database, only three alleles (*001, *003, *005) occur in our cohort of 426 healthy individuals (Fig. 2A). Phylogenetic analysis suggests that these three alleles typify separate functional groups (Supplemental Fig. 1B). Four ARMS PCR reactions define the three groups, and two supplemental reactions increase the resolution of the method (Table 1). With this approach, we can separate six different individual alleles or groups of alleles (*KIR2DL2*-G*001, -G*003, -G*005, *004, *006, *009) (Supplemental Fig. 2C,D).

All *KIR2DL2* reactions were optimized using DNA from 178 of the 426 healthy donors. Previously reported analysis of these same DNA samples could not completely resolve KIR2DL2*005 from KIR2DL2*001^23^. With our method, we did not identify the presence of KIR2DL2*005 and instead found that 21 of the 21 samples with ambiguous typing exhibited KIR2DL2*001. The presence of KIR2DL2*001 and the absence of KIR2DL2*005 in these samples were confirmed by sequencing by an independent laboratory (data not shown). Figure 2A displays the amino acid alignment of *KIR2DL2* alleles and segregation of alleles by the typing methodology. Ambiguity in interpretation for certain combinations of alleles occurs, as indicated (Fig. 2B).

### *KIR2DL3* allele typing

**Figure 3:**
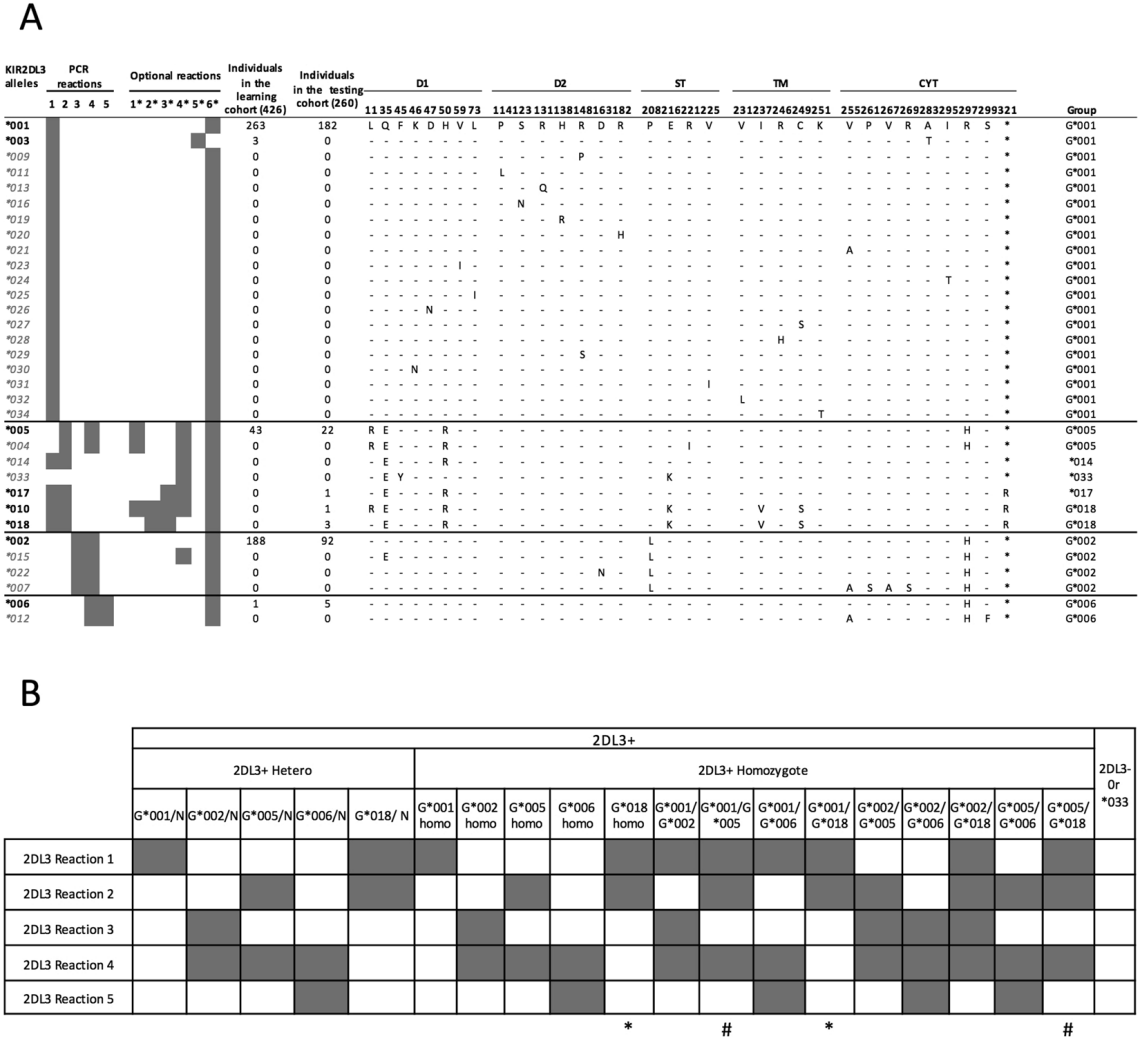
*KIR2DL3* allele typing method. (**A**) Alignment of the amino acid sequences of the 34 known *KIR2DL3* allelic variants. A dash indicates identity with the consensus KIR2DL3*001, an (*) indicates a stop codon. Structural domains are indicated: Ig-like domains (D1 and D2), stem domain (ST), transmembrane domain (TM), and cytoplasmic domain (CYT). Five PCR reactions separate the four subgroups identified by phylogenetic analysis. Six additional PCR reactions separate alleles within subgroups. Frequencies of the alleles present in the learning cohort of 426 individuals and in the testing cohort of 260 individuals are indicated. The group identification for each allele is indicated. The alleles tested by PCR are in bold black font, and the non-tested alleles are in gray italics. (**C**) *KIR2DL3* PCR interpretation guide. PCR profiles marked by * or # are similar and require a higher resolution of genotyping using supplemental reactions.

Of 34 *KIR2DL3* alleles published in an EMBL-EBI IPD *KIR* database, only five (*001, *002, *003, *005, *006) were identified in the cohort of 426 healthy individuals (Fig. 3A). Phylogenetic analysis (Supplemental Fig. 1C) suggested four separate groups, which could be segregated by five distinct main ARMS PCR reactions (Fig. 3B), complemented by six supplemental reactions to further optimize resolution. With these eleven PCR reactions (Table 1), we could separate eleven different alleles or groups of alleles (*KIR2DL3*-G*001, -G*002, -G*005, *003, *006, *009, *010, *014, *015, *017, *018) (Supplemental Fig. 1E,F).

We confirmed accuracy of the typing methods for *KIR2DL1*, *KIR2DL2*, and *KIR2DL3* by comparing results using this methodology for 200 donors of the validation cohorts to results generated by another laboratory by a sequence-based typing methodology, finding 99.6% concordance^26,27^.

### Linkage disequilibrium between *KIR2DL* alleles

**Figure 4:**
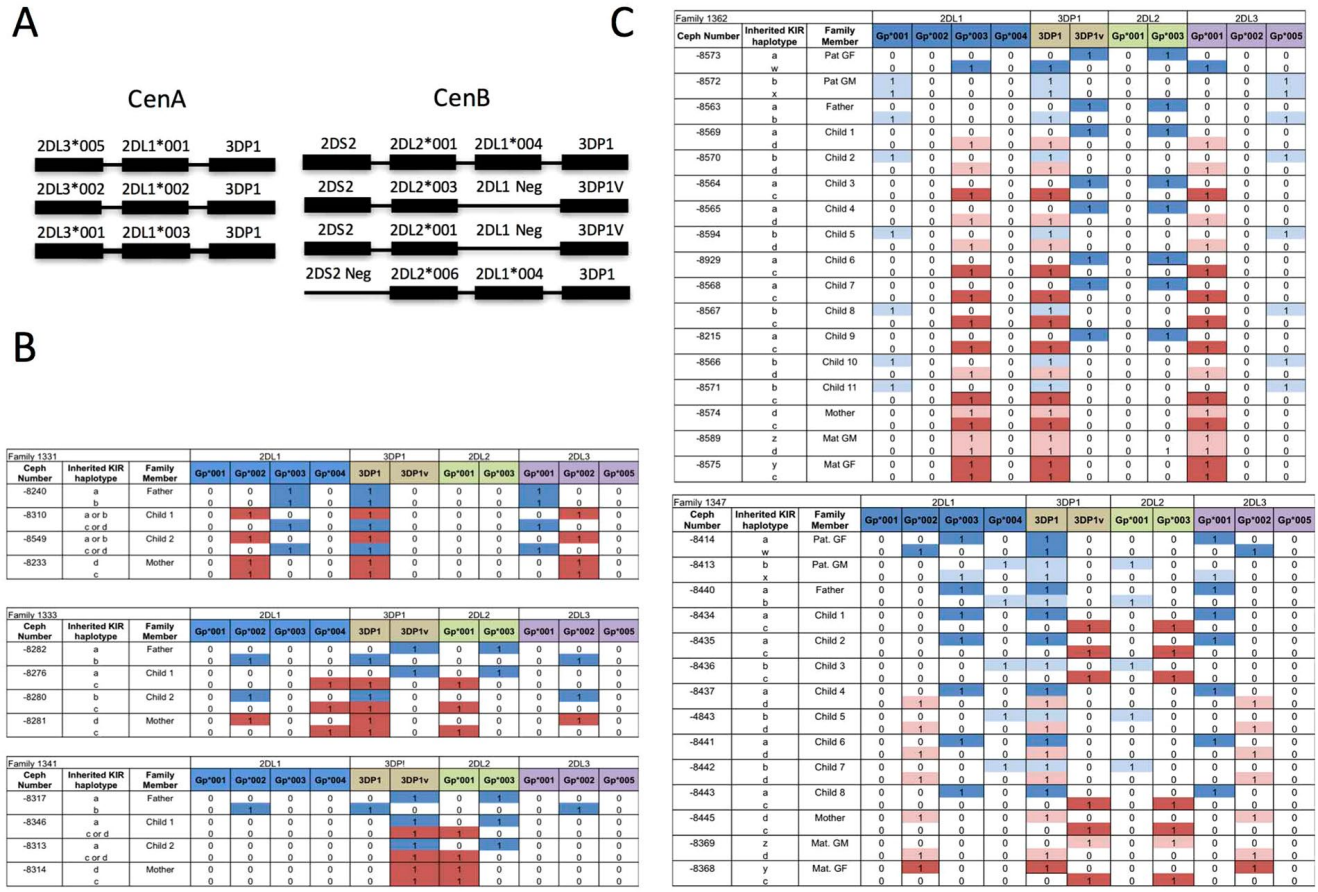
Linkage disequilibrium between KIR2DL alleles. (**A**) Linkage disequilibrium analysis identifies seven common combinations of centromeric *KIR2DL* alleles in a cohort of 260 individuals. (**B**) Allelic segregation of the *KIR2DL* and *KIR3DP1* alleles in CEPH families. Paternal *KIR2DL* alleles are shown in blue; maternal alleles in red. (**C**) *KIR2DL* allele typing from three generations of CEPH family individuals demonstrates Mendelian inheritance of allele combinations established by the LD study.

We calculated LD between alleles of *KIR2DL1*, *KIR2DL2*, *KIR2DL3*, *KIR3DP1*, and the presence or absence of gene *KIR2DS2* in 260 individuals (Fig. 4A). Seven allele combinations, three belonging to the canonical centromeric haplotype-A (*KIR2DL1-KIR2DL3-KIR3DP1*)^23,28^ and four belonging to the centromeric haplotype-B, were represented in more than 95% of the donors. Of interest, the centromeric haplotype-B allele combination comprised of KIR2DL1*004, KIR2DL2*006 and the absence of *KIR2DS2* was found in 2.3% of individuals typed.

To confirm the LD observed by calculation in our cohort, we also genotyped immortalized B cell lines from the Centre d’Etude Polymorphisme Humaine (CEPH). We selected 5 families and performed *KIR2DL* allele typing using our methodology (Fig. 4B,C). The typing results demonstrated a Mendelian inheritance of allele combinations established by the LD study.

## Discussion

We have established a comprehensive genotyping method to distinguish alleles and allele groups for the *KIRL2DL1*, *KIR2DL2* and *KIR2DL3* genes. We validated the methodology using 178 donors from a learning cohort and 260 samples from a validation cohort, further confirming its robustness by sequence-based typing. Designed as a typing kit for the centromeric region of the *KIR* haplotype, our methodology provides a reliable, cost-effective alternative to sequencing methods that can be employed using basic laboratory equipment.

The *KIR2DL1* typing method identifies the four most common *KIR2DL1* alleles, in addition to the less common KIR2DL1*006 and KIR2DL1*010 alleles found in three individuals. The learning cohort of 178 individuals was mostly comprised of Caucasian individuals, while the testing cohort was more ethnically diverse. For the 136 individuals in the testing cohort for whom we could obtain ethnicity, 59.6% were Caucasian,17.6% Asian, 16.2% African-American, 3.7% Hispanic, and 2.9% mixed ethnicity. Consistent with this population diversity, our typing revealed in several individuals the presence of KIR2DL1*006, an allele previously reported to be relatively well-represented (7%) in an African-American cohort^29^. With our *KIR2DL2* typing method, we can now faithfully resolve ambiguities between KIR2DL2*005 and KIR2DL2*001 reported with previous methodologies^23^. A third typing methodology validated our results^26,27^.

Copy number for the *KIR2DL1* alleles can be estimated using typing for the framework pseudogene *KIR3DP1*, where the KIR3DP1V (*001, *002, *004, *007, *009, *011, *012) alleles are associated with the absence of *KIR2DL1*. Concerning *KIR2DL2* and *KIR2DL3*, copy number estimation was based on the mutual non-co-expression due to the allelic relationship of the two genes for the same locus. Our method has its limitations and is accurate for content only with copy number and haplotype inferred by LD. It has previously been reported that an additional *KIR2DL1*, *KIR2DL2* or *KIR2DL3* allele occurs in 1 to 2% of the population^30^. To identify these individuals, a quantitative PCR assay would be informative in addition to our typing method to calculate more accurately copy number of each *KIR* allele^31^.

The LD analysis identified the seven predominant combinations of *KIR2DL* receptors, representing more than 95% of the 260 donors studied. Genotyping of the CEPH family further validated the utility of our method and confirmed the Mendelian inheritance of the allele combinations established by the LD study.

This typing methodology will facilitate future studies aimed at determining if functional differences exist between alleles, as suggested by phylogenetic segregation. As has previously been demonstrated for the *KIR3DL1* alleles, diversity in cell surface expression, ligand affinity, and effector function for the *KIR2DL* alleles may combine to modulate NK education and influence innate immune response to viral pathogens and malignancy.

## Methods

### Genomic analyses and Primer design

All allele-coding sequences of *KIR2DL1, KIR2DL2* and *KIR2DL3* from the EMBL-EBI IPD KIR database sequences (http://www.ebi.ac.uk/ipd/kir/alleles.html) were included in our alignment analyses. We performed gene alignments and phylogenetic analyses using MacVector software version 13.5.5. Protein sequences of alleles for *KIR2DL1* (Supplemental Fig. 1A), *KIR2DL2* (Supplemental Fig. 1B) and *KIR2DL3* (Supplemental Fig. 1C) were aligned and analyzed by tree building methods: neighbor joining (Uncorrected method, Best Tree) with MacVector. Genomic sequencing in a cohort of 426 European-American healthy donors previously identified nine *KIR2DL1*, three *KIR2DL2* and five *KIR2DL3 alleles* respectively^23^. Among *KIR2DL1*, *KIR2DL2*, and *KIR2DL3* alleles, four, three, and three alleles were found with >1% frequency respectively. Sequence homology was then used in conjunction with the phylogenetic analyses to categorize alleles into *KIR* allele subgroups, for which PCR primer combinations were then designed. Low frequency alleles were assigned to subgroups based on sequence homology in the exon coding regions. Primer pairs targeting SNPs present in each subtype group were identified and their specificity for *KIR2DL1, KIR2DL2* or *KIR2DL3* was confirmed using NCBI primer blast. To provide an internal control for DNA quality, an 813 bp control band derived from a conserved region of the *APC* gene was multiplexed into each reaction. Specific primer sequences and PCR conditions are shown in Table 1. The position of the SNP targeted is based on the following genomic sequences: for *KIR2DL1* primers KIR2DL1*00303 (IPD Acc No: KIR00005), for *KIR2DL2* primers KIR2DL2*0030101 (IPD Acc No: KIR00012), for *KIR2DL3* primers KIR2DL3*0010101 (IPD Acc No: KIR00014).

For *KIR2DL1*, we designed six PCR reactions to delineate six distinct allele groups based on the coding sequences and four supplemental reactions to identify additional subgroups or individual alleles represented in the n = 426 cohort (Fig. 1A). We designed one additional reaction for *KIR3DP1-3DP1V* to determine *KIR2DL1* copy number^5^. For *KIR2DL2* alleles, we designed four PCR reactions to separate three distinct groups with two supplemental reactions to identify subgroups (Fig. 2A). For *KIR2DL3* alleles, five PCR reactions separate alleles into four distinct groups, with six supplemental reactions to identify some subgroups or individual alleles (Fig. 3A). The design of the primers was optimized using the software AmplifX (V1.7.0, http://crn2m.univ-mrs.fr/pub/recherche/equipe-t-brue/jullien-nicolas/programmation/amplifx/), following the principles of amplification refractory mutation system (ARMS)-PCR^32^. All primers are ARMS-PCR primers, with the exceptions of the control primers and *KIR2DL1* allele primer pair #7, and were designed for an annealing temperature of 63 °*C* (Table 1). We used a testing cohort of 260 healthy individuals whose KIR genotypes had been identified by sequence-based typing to verify the specificity of the primers, as well as 178 DNA from the European-American healthy donors (Figs 1A–3A).

### PCR Reactions

The ProFlex PCR system (Life Technologies) was used to optimize and validate the PCR reaction conditions. Each 20 µL reaction included 50–100 ng of DNA and was prepared with Taq polymerase (0.25 µL), dNTP (0.5 µL) and PCR buffer (2 µL) (Roche). Each primer was used at a final concentration of 0.5 µM. All reactions used the following PCR template: 95 °*C* 5 min, (95 °*C* 15 s, 63 °*C* 20 s, 72 °*C* 1 min) X 40 cycles, 72 °*C* 7 min, with the exception of 2DL2 PCR reaction #4, which utilized the following conditions: 95 °*C* 5 min, (95 °*C* 15 s, 63 °*C* 20 s, 72 °*C* 2.5 min) X 40 cycles, 72 °*C* 7 min. (Control primers were designed to amplify a fragment of the *APC* gene. All reactions utilize reaction-specific primers and the control primers, except for *KIR2DL1* PCR reaction 7 and *KIR2DL2* PCR reaction 4, which do not include control primers. We analyzed all PCR products using electrophoresis on 1.5% agarose gels for 40 min at 125 V. Control bands (813 bp) confirmed DNA quality. Specific product sizes ranged from 0.2–2.3 kb (Table 1)

### PCR interpretation

The *KIR2DL1, KIR2DL2* and *KIR2DL3* PCR profiles are displayed respectively in Figs 1B–3B. In cases where the observed results prompt multiple interpretations, supplemental reactions can be used to increase resolution. A few very rare allelic combinations cannot be discerned from each other and are indicated as ambiguous combinations in the table footnotes. Gel electrophoresis and interpretation of the basic and supplemental reactions for *KIR2DL1*, *KIR2DL2*, and *KIR2DL3* are shown in Supplemental Fig. 2.

### Cells, DNA Sources and Preparation

Genomic DNA was extracted from cell lines, frozen peripheral blood mononuclear cells (PBMC) and whole blood using blood mini kits according to the manufacturer’s instructions (Qiagen). EBV-immortalized cell lines derived from multi-generational families were produced by the Centre d’Etude Polymorphisme Humaine (CEPH) (http://www.cephb.fr/en/familles_CEPH.php-presentation). Samples were anonymized by the CEPH. DNA samples from unrelated hematopoietic stem cell donors were collected under National Marrow Donor Program (NMDP) Institutional Review Board-informed research consent and provided by the NMDP Research Repository. We collected PBMC from consenting healthy human donors at MKSCC and the New York Blood Center, following approval from the MSKCC Institutional Review Board. Additional PBMC were isolated from buffy coats obtained from healthy volunteer donors via the New York Blood Center (http://nybloodcenter.org/). The MSKCC IRB waived the need for additional research consent for anonymous NYBC samples.

### Statistics

Linkage disequilibrium (LD) between pairs of *KIR* alleles was calculated according to Mattiuz *et al*.^33^, and the significance of LD values was assessed by χ^2^ analysis.

### Statement of Informed consent

All methods were performed in accordance with the relevant guidelines and regulations. Informed consent was obtained from all individual participants included in the study.

## Electronic supplementary material

Supplementary Figures
