# Publisher Correction: Novel multiplex PCR-SSP method for centromeric *KIR* allele discrimination

**DOI:** 10.1038/s41598-019-43449-3

**Published:** 2019-10-18

**Authors:** Jean-Benoît Le Luduec, Anupa Kudva, Jeanette E. Boudreau, Katharine C. Hsu

**Affiliations:** 1 Immunology Program, Sloan-Kettering Institute for Cancer Research, New York, NY USA; 2 Department of Pediatrics, Memorial Sloan Kettering Cancer Center, New York, NY USA; 3 Department of Medicine, Weill Cornell Medical College, New York, NY USA; 4 Department of Medicine, Memorial Sloan Kettering Cancer Center, New York, NY USA; 5 Present Address: Departments of Pathology and Microbiology & Immunology, Dalhousie University, Halifax, Canada

Correction to: *Scientific Reports* 10.1038/s41598-018-33135-1, published online 05 October 2018

In the original version of this Article, the author Jean-Benoît Le Luduec was incorrectly indexed. This error has now been corrected.

