# Supplementary material for: Novel multiplex PCR-SSP method for centromeric KIR allele discrimination
Source: Sci Rep. 2018 Oct 5;8:14853. doi: 10.1038/s41598-018-33135-1 (PMC6173694; doi:10.1038/s41598-018-33135-1)
Supplement: Supplementary file 1 — Supplementary Figures [file 41598_2018_33135_MOESM1_ESM.pdf]

Novel multiplex PCR-SSP method for centromeric *KIR* allele discrimination

Jean-Benoît Le Luduec, Anupa Kudva, Jeanette E. Boudreau, Katharine C. Hsu

SUPPLEMENTAL FIGURES

Supplemental Figure 1: Phylogenetic studies of KIR variants amino acid sequences.

(A) Phylogenetic study of *KIR2DL1* allotypes. (B) Phylogenetic study of *KIR2DL2* allotypes. (C) Phylogenetic study of *KIR2DL3* allotypes. The different groups defined are highlighted in gray.

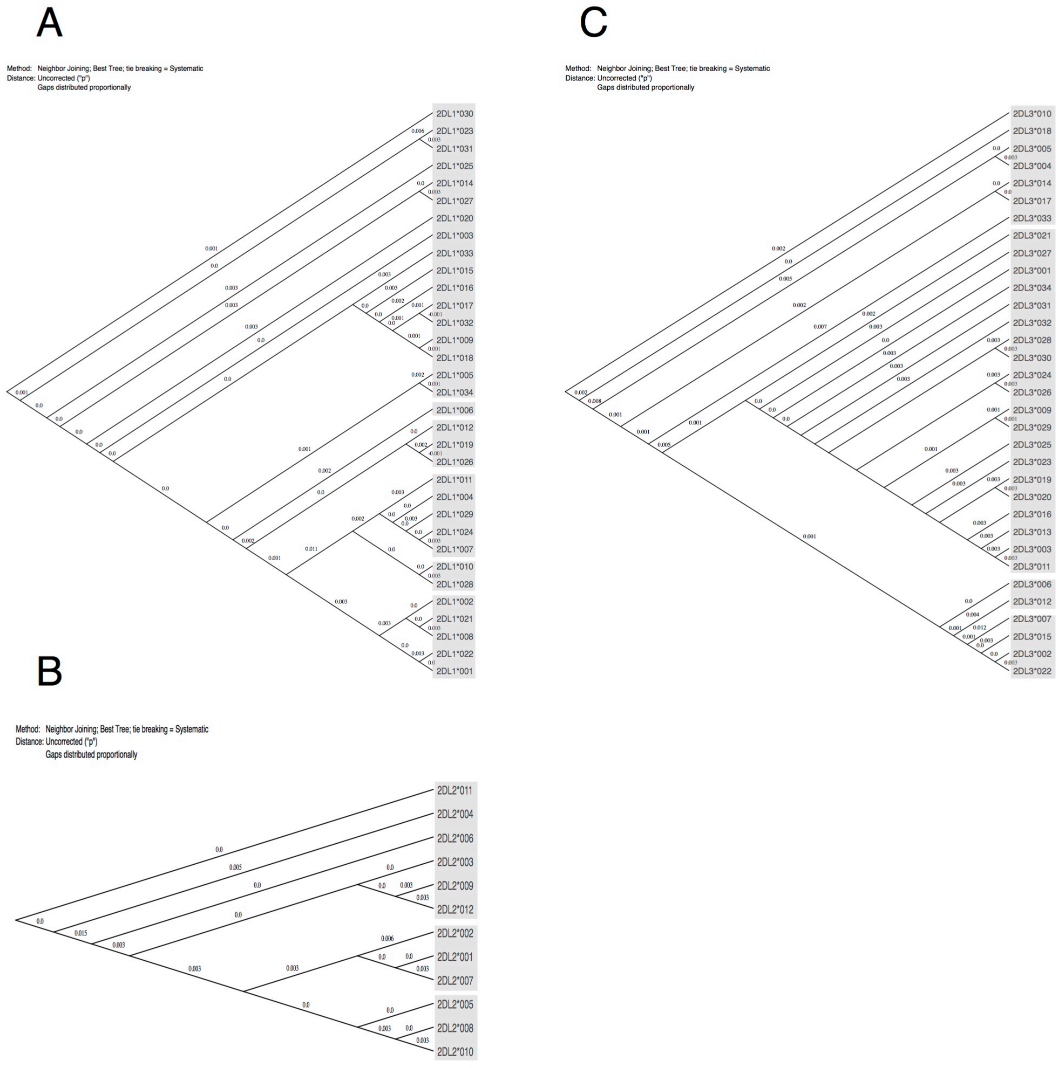

Supplemental Figure 2: Electrophoresis gels of *KIR2DL* allele typing

The figures are made grouping cropped images of gels from different experiments. All the original gels with time of exposure indicated are available in supplement material. (A) Electrophoresis gels of the six basic *KIR2DL1* PCR reactions. An additional reaction (R7) genotype, the pseudo-genes *KIR3DP1* (Higher band), and *KIR3DP1V* (Lower band) identify the copy number of *KIR2DL1*. (B) Electrophoresis gel of the eleven *KIR2DL1* PCR reactions, including the four supplemental PCR reactions, which increase typing resolution. (C) Electrophoresis gels of the four basic *KIR2DL2* PCR reactions. (D) Electrophoresis gels of the six *KIR2DL2* PCR reactions, including the two supplemental PCR reactions. (E) Electrophoresis gels of the five basic *KIR2DL3* PCR reactions. (F) Electrophoresis gel of the eleven *KIR2DL3* PCR reactions, including the six supplemental PCR reactions.

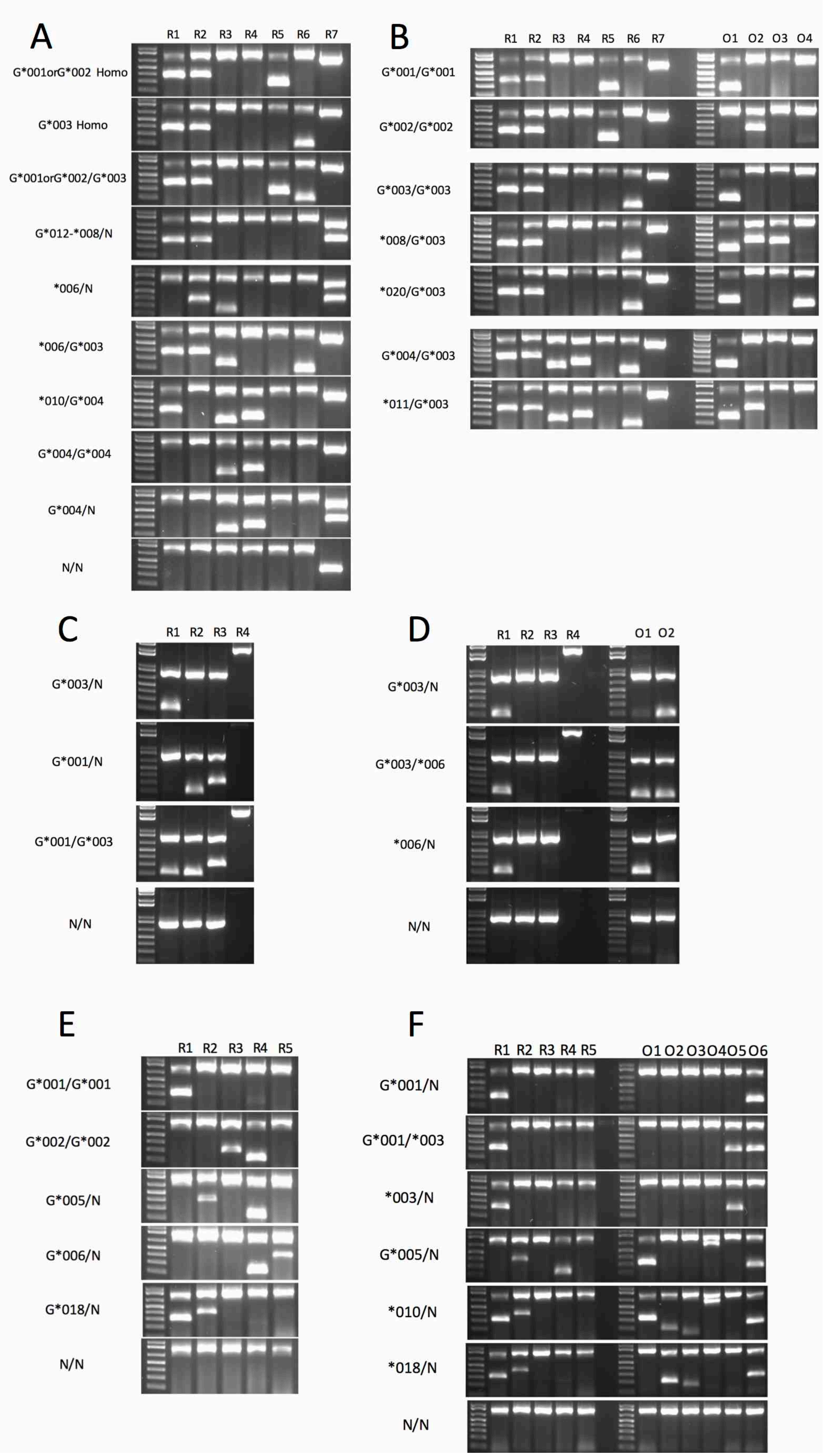
A
R1 R2 R3 R4 R5 R6 R7
G*001orG*002 Homo
G*003 Homo
G*001orG*002/G*003
G*012-*008/N
*006/N
*006/G*003
*010/G*004
G*004/G*004
G*004/N
N/N
B
R1 R2 R3 R4 R5 R6 R7
O1 O2 O3 O4
G*001/G*001
G*002/G*002
G*003/G*003
*008/G*003
*020/G*003
G*004/G*003
*011/G*003
C
R1 R2 R3 R4
G*003/N
G*001/N
G*001/G*003
N/N
D
R1 R2 R3 R4
O1 O2
G*003/N
G*003/*006
*006/N
N/N
E
R1 R2 R3 R4 R5
G*001/G*001
G*002/G*002
G*005/N
G*006/N
G*018/N
N/N
F
R1 R2 R3 R4 R5
O1 O2 O3 O4 O5 O6
G*001/N
G*001/*003
*003/N
G*005/N
*010/N
*018/N
N/N
